# Characterization of a *Deinococcus radiodurans* MazF: A UACA‐specific RNA endoribonuclease

**DOI:** 10.1002/mbo3.501

**Published:** 2017-07-03

**Authors:** Tatsuki Miyamoto, Yuri Ota, Akiko Yokota, Tetsushi Suyama, Satoshi Tsuneda, Naohiro Noda

**Affiliations:** ^1^ Department of Life Science and Medical Bioscience Waseda University Tokyo Japan; ^2^ Biomedical Research Institute National Institute of Advanced Industrial Science and Technology Ibaraki Japan

**Keywords:** *Deinococcus radiodurans*, MazEF, sequence specificity, toxin–antitoxin system

## Abstract

Microbes are known to withstand environmental stresses by using chromosomal toxin–antitoxin systems. MazEF is one of the most extensively studied toxin–antitoxin systems. In stressful environments, MazF toxins modulate translation by cleaving single‐stranded RNAs in a sequence‐specific fashion. Previously, a chromosomal gene located at DR0417 in *Deinococcus radiodurans* was predicted to code for a MazF endoribonuclease (MazF_DR__0417_); however, its function remains unclear. In the present study, we characterized the molecular function of MazF_DR__0417_. Analysis of MazF_DR__0417_‐cleaved RNA sites using modified massively parallel sequencing revealed a unique 4‐nt motif, UACA, as a potential cleavage pattern. The activity of MazF_DR__0417_ was also assessed in a real‐time fluorometric assay, which revealed that MazF_DR__0417_ strictly recognizes the unique tetrad UACA. This sequence specificity may allow *D. radiodurans* to alter its translation profile and survive under stressful conditions.

## 1 INTRODUCTION

Toxin–antitoxin (TA) systems are common in the bacterial and archaeal kingdoms (Pandey & Gerdes, 2005). They are typically encoded by operons comprising the genes of a stable toxin that disrupts cellular activities and an unstable antitoxin that alleviates the toxin's effect. In response to stress, antitoxins are rapidly degraded because of their labile nature, thus freeing the corresponding toxins (Page & Peti, 2016). These toxins then arrest prokaryotic growth in many different ways; for example, they can impede DNA replication, cell wall synthesis, translation, cell division, and ATP production (Schuster & Bertram, 2013). Among these mechanisms, translation inhibition caused by intracellular RNA digestion is the most common (Yamaguchi & Inouye, 2011).

The MazEF family, which is comprised of MazE antitoxin and MazF toxin, is one such representative TA system (Aizenman, Engelberg‐Kulka, & Glaser, 1996). In *Escherichia coli*, MazE forms a heterohexamer with MazF and inactivates the activity of MazF as a sequence‐specific endoribonuclease (Kamada, Hanaoka, & Burley, 2003). Once cells encounter specific stresses, however, unstable MazE is preferentially degraded by ClpAP protease, liberating the MazF toxin endoribonuclease (Aizenman et al., 1996; Hazan, Sat, & Engelberg‐Kulka, 2004). The released MazF then alters translation globally by cutting cellular RNAs at ACA sites (Amitai, Kolodkin‐Gal, Hananya‐Meltabashi, Sacher, & Engelberg‐Kulka, 2009; Sauert et al., 2016; Vesper et al., 2011; Zhang et al., 2003). Although the functions of prokaryotic MazF toxins remain unclear, they are thought to have various biological roles such as in virulence (Rothenbacher et al., 2012; Tiwari et al., 2015; Zhu et al., 2009), phage defense systems (Alawneh, Qi, Yonesaki, & Otsuka, 2016; Hazan & Engelberg‐Kulka, 2004), persister generation (Tripathi, Dewan, Siddique, & Varadarajan, 2014), and programmed cell death (Nariya & Inouye, 2008); an important characteristic of MazF endoribonucleases that may contribute to these physiological functions is their sequence‐specificities. In most cases, MazF toxins require strict sequences for RNA cleavages, which are typically 3–7‐nucleotide (nt) motifs (Miyamoto, Kato, Sekiguchi, Tsuneda & Noda, 2016; Miyamoto, Yokota, Tsuneda, Noda et al. 2016; Nariya & Inouye, 2008; Rothenbacher et al., 2012; Schifano et al., 2014; Schuster et al., 2013; Verma & Bhatnagar, 2014; Yamaguchi & Inouye, 2011; Yamaguchi, Nariya, Park, & Inouye, 2012; Zhang et al., 2003; Zhu et al., 2006, 2008, 2009). Thus, microbes may reprogram their translation by shutting down most translation processes or by eliminating specific transcripts to cope with unfavorable surroundings.

*Deinococcus radiodurans* is a Gram‐positive bacterium that inhabits a variety of environments. It is remarkably resistant to different types of stress, such as desiccation, oxidative stress, DNA damage, ionizing radiation, and ultraviolet radiation (Makarova et al., 2001). Previously, computational analysis predicted that a chromosomal gene, located at DR0417 in *Deinococcus radiodurans*, codes for a MazF toxin (MazF_DR0417_) (Chopra, Saumitra, Pathak, Bhatnagar, & Bhatnagar, 2013; Pandey & Gerdes, 2005), suggesting that *D. radiodurans* utilizes MazF_DR0417_ as a posttranscriptional regulator and regulates its translation under stressful conditions. In the present study, we found that MazF_DR0417_ is a toxin endoribonuclease and constitutes an authentic TA system together with its cognate antitoxin MazE, encoded by the locus DR0416 (MazE_DR0416_). Analysis of MazF_DR0417_‐cleaved RNA products using modified massively parallel sequencing revealed that MazF_DR0417_ cleaves RNA specifically at a unique 4‐nt motif, UACA. The indispensability of the tetrad for effective RNA cleavage was also demonstrated using a fluorescent quenching approach. The results indicate that MazF_DR0417_ may play a role in adaptation to stressful environments by promoting the selective degradation of intracellular RNAs.

## 2 MATERIALS AND METHODS

### 2.1 Plasmids and oligonucleotides

The expression vector pET21c was purchased from Takara Bio Service (Shiga, Japan). The pET19b expression vector encoding *mazE*_DR0416_, whose codon usage was optimized for recombinant protein expression in *E. coli*, was purchased from GenScript Japan (Tokyo, Japan). Double‐stranded DNA fragments, including the *mazF*_DR0417_ DNA sequence flanked by *Bam*HI/*Eco*RI sites, were purchased from Life Technologies Japan Ltd. (Tokyo, Japan). The pMX‐T vector encoding *D. radiodurans mazEF* genes was purchased from Life Technologies Japan Ltd. Fluorescently modified oligonucleotides were purchased from Japan Bio Services (Saitama, Japan). The chemically synthesized tRNA^Val^ oligonucleotide was purchased from Gene Design (Osaka, Japan).

### 2.2 Plasmid construction

Double‐stranded DNA fragment encoding *mazF*_DR0417_ gene was digested with *Eco*RI and *Bam*HI (Toyobo, Osaka, Japan) and purified using a MinElute PCR purification kit (Qiagen, Hilden, Germany). Likewise, the pMX‐T plasmid encoding *D. radiodurans mazEF* genes (*mazEF*_DR0416DR0417_) was digested with *Bam*HI (New England Biolabs, Ipswich, MA, USA) and cleaned with a QIAquick PCR purification kit (Qiagen). The linearized pMX‐T plasmid was then digested with *Eco*RI (Takara), and the DNA fragment containing *mazEF*_DR0416DR0417_ genes was recovered by using a QIAquick gel extraction kit (Qiagen). These DNA fragments were then ligated into the corresponding pET21c multiple cloning site using a DNA ligation kit (Takara), producing pET21c‐*mazF*_DR0417_ and pET21c‐*mazEF*_DR0416DR0417_, and they were introduced into the *E. coli* strain DH5α (Nippon Gene, Tokyo, Japan). pET21c‐*mazF*_DR0417_ and pET21c‐*mazEF*_DR0416DR0417_ were extracted using a QIAprep Spin Miniprep Kit (Qiagen) and the sequences were validated using an AB 3500 Genetic Analyzer (Applied Biosystems, Foster City, CA, USA), according to the manufacturer's protocol.

### 2.3 Growth effect of MazE_DR0416_ and MazF_DR0417_

The *E. coli* cells harboring pET21c empty vector, pET21c encoding *D. radiodurans mazF* or *mazEF* were cultivated at 37°C for 12 h in liquid LB medium supplemented with 100 μg/mL ampicillin. Turbid overnight cultures were then streaked onto the LB plates containing 100 μg/mL ampicillin and 0.2% glucose with or without 25 μmol/L IPTG at 37°C.

### 2.4 Protein expression

pET19b‐*mazE*_DR0416_ was introduced into the *E. coli* strain BL21 (DE3) (BioDynamics Laboratory Inc., Tokyo, Japan), whereas pET21c‐*mazF*_DR0417_ was introduced into the strain BL21 (DE3) (Nippon Gene) using the heat‐shock method. The *E. coli* cells harboring pET19b‐*mazE*_DR0416_ or pET21c‐*mazF*_DR0417_ were grown overnight in liquid LB medium supplemented with 100 μg/mL ampicillin at 37°C. These cells were inoculated into 1 L LB medium containing 100 μg/mL ampicillin. One millimolar IPTG was added to induce MazE_DR0416_ and MazF_DR0417_ when the OD_600_ reached 0.8 and 3.0, respectively. The cells were harvested by centrifugation at 7,000*g* after 5 and 3.5 h of incubation for MazE_DR0416_ and MazF_DR0417_, respectively.

### 2.5 Purification of MazE_DR0416_

*Escherichia coli* cells containing MazE_DR0416_ were thawed on ice and suspended in 18 mL of 6 mol/L urea buffer (10 mmol/L sodium phosphate buffer (pH 8.0), 150 mmol/L NaCl, 0.025% Triton X‐100, 6 mol/L urea, 2.5 mmol/L β‐mercaptoethanol, and 20 mmol/L imidazole). The suspended cells were then incubated on ice for 5 min in the presence of 0.09 mg/mL lysozyme. The cells were lysed by sonication and collected by centrifuging at 7000*g*. The supernatant was then filtered through a 0.45‐μm membrane (Millex, Darmstadt, Germany) and applied to a 1‐mL His‐Trap FF column (GE Healthcare, Little Chalfont, UK). Urea was then removed by gradually substituting the 6 mol/L urea buffer with binding buffer (20 mmol/L sodium phosphate buffer (pH 8.0), 300 mmol/L NaCl, 0.05% Triton X‐100, 5 mmol/L β‐mercaptoethanol, and 40 mmol/L imidazole) using an AKTA pure 25 (GE Healthcare). The following program was used for this procedure: flow rate, 1 mL/min; linear elution gradient, 20 column volumes (cv). Subsequently, the column was washed with 32 cv of binding buffer. Deca‐histidine‐tagged MazE_DR0416_ was selectively eluted by increasing the concentration of elution buffer (20 mmol/L sodium phosphate buffer (pH 8.0), 300 mmol/L NaCl, 0.05% Triton X‐100, 5 mmol/L β‐mercaptoethanol, and 500 mmol/L imidazole) using the following program: flow rate, 1 mL/min; linear elution gradient, 20 cv; fraction size, 0.5 mL. Molecular weight and purity were confirmed by the Agilent 2200 TapeStation P200 ScreenTape Assay (Agilent Technologies, Santa Clara, CA, USA). Protein concentration was determined using the Qubit Protein Assay Kit (Life Technologies, Carlsbad, CA, USA).

### 2.6 Purification of MazF_DR0417_

The recombinant MazF_DR0417_ was purified as described previously (Miyamoto et al. 2016, 2016), with slight modifications. *Escherichia coli* cells containing MazF_DR0417_ were thawed on ice and suspended in 32 mL of binding buffer (20 mmol/L sodium phosphate buffer (pH 8.0), 300 mmol/L NaCl, 5 mmol/L β‐mercaptoethanol, and 50 mmol/L imidazole). The cells were lysed by sonication and collected by centrifuging at 7000 *g*. The supernatant was then filtered through a 0.45‐μm membrane (Millex) and applied to a 1‐mL His‐Trap FF crude column (GE Healthcare). Nonspecifically bound proteins were removed by washing with 32 cv of binding buffer using AKTA pure 25 (GE Healthcare). Hexa‐histidine‐tagged MazF_DR0417_ was selectively eluted by gradually increasing the elution buffer concentration using the following program: flow rate, 1 mL/min; linear elution gradient, 20 cv; fraction size, 0.5 mL. The composition of the elution buffer was as follows: 20 mmol/L sodium phosphate buffer (pH 8.0), 300 mmol/L NaCl, 5 mmol/L β‐mercaptoethanol, and 500 mmol/L imidazole. Molecular weight and purity were confirmed by the Agilent 2200 TapeStation P200 ScreenTape Assay (Agilent Technologies). Protein concentration was determined using the Qubit Protein Assay Kit (Life Technologies).

### 2.7 Enzymatic activity of MazE_DR0416_ and MazF_DR0417_

Synthetic RNA constructs were prepared as described in our previous study (Miyamoto et al. 2016). One hundred nanograms of RNA 500‐2 was incubated with 1.2, 6, or 30 pmol of MazF_DR0417_ at 37°C for 2 h in MazF reaction buffer (20 mmol/L Tris‐HCl (pH 8.0), 1 mmol/L dithiothreitol, 0.01% Triton X‐100, and 4 U of recombinant RNase inhibitor (Takara)) in a 50‐μL reaction volume. As a control reaction, 30 pmol of MazF_DR0417_ was preincubated with 300 pmol of MazE_DR0416_ at room temperature for 10 min, and this mixture was incubated with 100 ng of RNA 500‐2 at 37°C for 2 h in MazF reaction buffer in a final volume of 50 μL. These RNAs were purified with RNA Clean and Concentrator^™^‐5 (Zymo Research, Orange, CA, USA). Next, gel loading buffer II (Ambion, Austin, TX, USA) was added to each sample. The samples were incubated at 95°C for 5 min and then separated on a 10% polyacrylamide gel containing 7 mol/L urea. The RNA was stained using SYBR Gold (Life Technologies) and then detected using a Typhoon 9210 imager (GE Healthcare).

### 2.8 Cleavage sequence identification

A sequencing library was constructed as described in our previous study (Miyamoto et al. 2016), with slight modifications. Briefly, five RNA mixtures were incubated with 1.5 μg of MazF_DR0417_ at 37°C for 30 min in MazF reaction buffer in a 20‐μL reaction volume. Phosphorylation, barcode ligation, and sequencing were performed as described previously (Miyamoto et al. 2016). The sequence data were analyzed with CLC Genomics 7.5.1., using the same parameters outlined in our previous work (Miyamoto et al. 2016). Relative coverage increase, which is defined as the coverage at the (n + 1)^th^ position divided by the coverage at the n^th^ position, was calculated for all reference samples. Nucleotide positions with coverage <500 were excluded from analysis. Of these nucleotide positions, those showing the overall top 15 and top 25 relative coverage increases were selected. The sequences five‐base pairs upstream and downstream of these positions were extracted and aligned using WebLogo (Crooks, Hon, Chandonia, & Brenner, 2004). These sequence data have been submitted to the DDBJ database under the accession number DRA004579.

### 2.9 Fluorometric analysis

Fluorometric analysis was performed as described previously (Miyamoto et al., 2016, 2016). Fifteen or 600 ng of MazF_DR0417_ was incubated with 10 pmol of fluorescently labeled oligonucleotides in MazF reaction buffer in a total volume of 20 μL. For control reactions, the oligonucleotides were also treated with 100 ng of RNase A (Novagen, Madison, WI, USA) in MazF reaction buffer in a final volume of 20 μL. All reactions were conducted at 37°C in triplicate. The fluorescence intensity was recorded every 1 min using a Light Cycler 480 system (Roche, Basel, Switzerland) with 483 nm excitation and 533 nm detection filters.

### 2.10 tRNA cleavage

Five picomoles of chemically synthesized tRNA^Val^ was incubated with 0.1, 0.9, or 8.1 pmol of MazF_DR0417_ at 37°C for 30 min in MazF reaction buffer in a 20‐μL reaction volume. Gel loading buffer II (Ambion) was added to each sample. These samples were incubated at 95°C for 5 min and then separated on a 10% polyacrylamide gel containing 7 mol/L urea. The RNA was stained using SYBR Gold (Life Technologies) and then detected using a Typhoon 9210 imager (GE Healthcare).

### 2.11 Accession numbers

The GenBank accession numbers are as follows: MazE_DR0416_ (NP_294139), MazF_DR0417_ (NP_294140), tRNA^Val^ (AE000513), and artificially designed RNAs 500‐2 (AB610940), 1000‐1 (AB610944), 1000‐2 (AB610945), 1000‐3 (AB610946), 1000‐4 (AB610947), and 1000‐5 (AB610948).

## 3 RESULTS

### 3.1 MazE_DR0416_ and MazF_DR0417_ constitute a TA system

**Figure 1:**
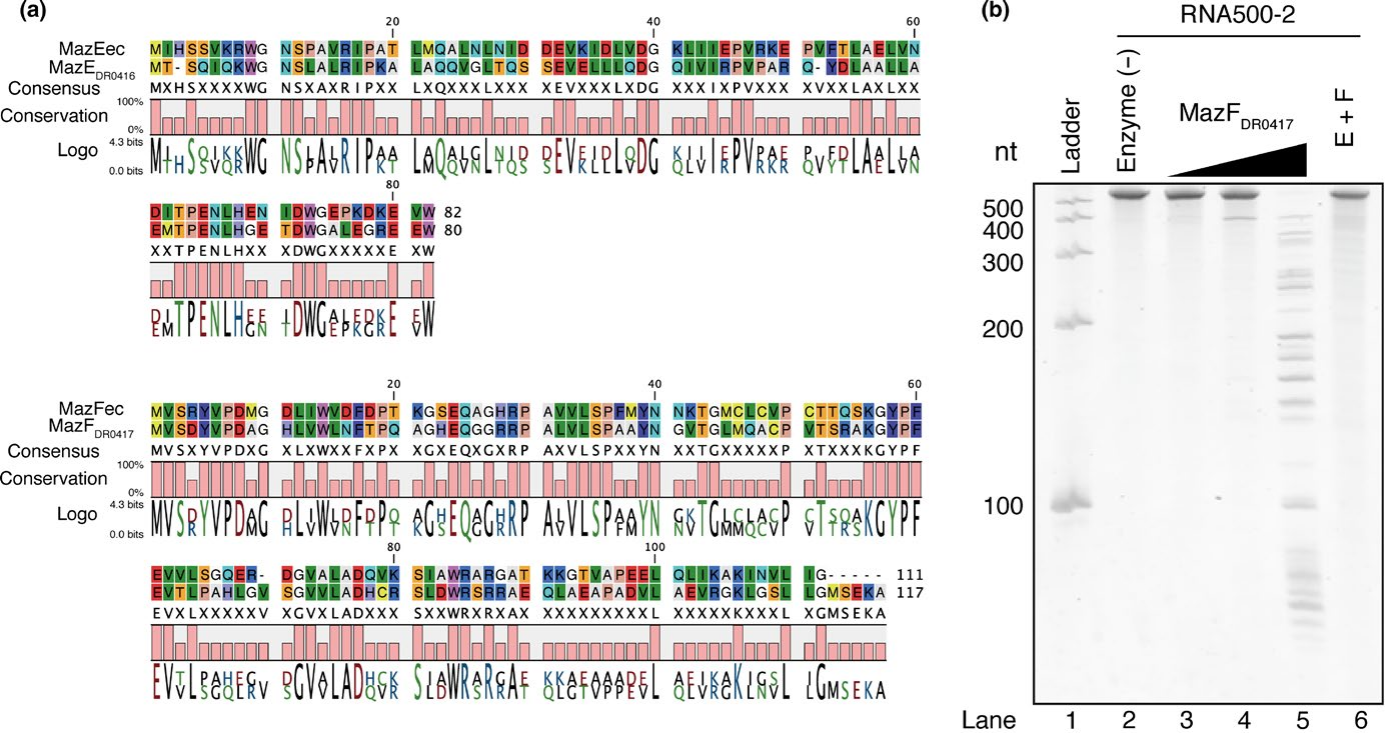
MazF endoribonuclease conserved in *D. radiodurans*. (a) Pairwise alignment of *E. coli* MazE (MazEec) and *D. radiodurans* MazE (MazE_DR__0416_) (upper panel); pairwise alignment of *E.coli* MazF (MazFec) and *D. radiodurans* MazF (MazF_DR__0417_) (lower panel). (b) MazF_DR__0417_‐mediated RNA cleavage. A 533‐nt artificially designed RNA (RNA 500‐2) was incubated with 1.2, 6, and 30 pmol of MazF_DR__0417_. The rightmost lane included 30 pmol of MazF_DR__0417_ preincubated with 300 pmol of MazE_DR__0416_

Previously, the genes located at DR0416 and DR0417 were presumed to encode a TA pair, the antitoxin MazE (MazE_DR0416_) and the toxin MazF (MazF_DR0417_) (Figure S1a), which shows 42.7% and 43.6% similarity to *E. coli* MazE and MazF, respectively (Figure 1a) (Chopra et al., 2013; Pandey & Gerdes, 2005). To briefly assess whether these components constitute a genuine TA system, we first cloned these genes into IPTG inducible vectors and expressed them in *E. coli*. As shown in Figure S1b, *E. coli* growth was inhibited when MazF_DR0417_ was expressed. In contrast, the cell growth was restored in the case of MazE_DR0416_ coexpression (Figure  S1b, right panel). Unexpectedly, we observed MazF_DR0417_‐mediated growth inhibition even in the absence of IPTG (Figure  S1b, middle panel); this would be probably because the ‘leaky’ expression of MazF_DR0417_ is toxic to the cells. We next purified the recombinant proteins to study the cleavage activity of MazF_DR0417_. When we investigated the purity and molecular weight of the protein by gel electrophoresis, a single peak was observed (Figure  S1c, lower panel), indicating that we had obtained highly purified MazF_DR0417_. We next coincubated MazF_DR0417_ with substrate RNA (RNA 500‐2), and dose‐dependent RNA fragmentation was observed (Figure 1b). Furthermore, the banding patterns of cleaved RNA were unique in length (Figure 1b, lane 5), indicating that MazF_DR0417_ is a functional toxin endoribonuclease possessing sequence specificity. Next, we purified MazE_DR0416_ (Figure  S1c, upper panel) and examined the effect of MazE_DR0416_ on quenching of the enzymatic activity of MazF_DR0417_. As expected, MazF_DR0417_‐catalyzed cleavage was abolished by preincubation with MazE_DR0416_ (Figure 1b, lane 6). Therefore, MazE_DR0416_ and MazF_DR0417_ constitute an authentic TA system.

### 3.2 MazF_DR0417_ recognizes specific 4‐nt motifs

**Figure 2:**
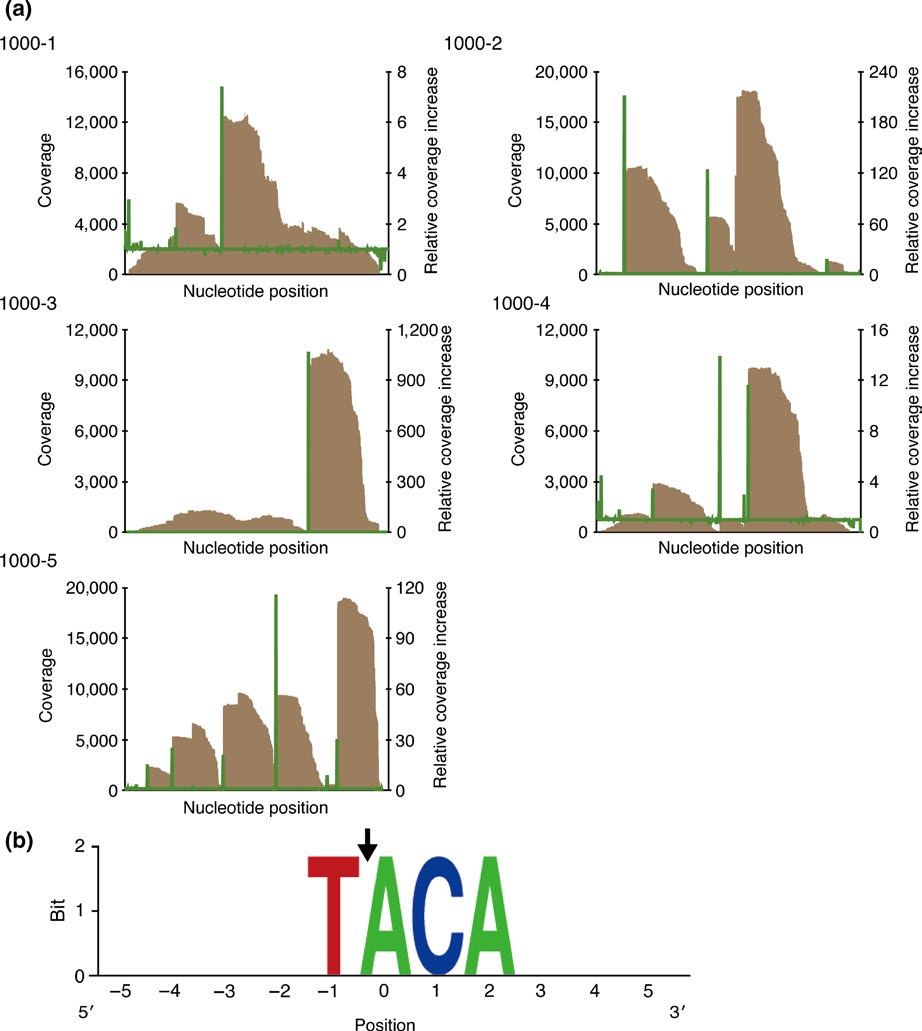
Analysis of the cleavage sequence of MazF_DR__0417_. (a) Graph of the coverage (brown bar) and relative coverage increase (green line). (b) Conserved sequences around the nucleotide positions with increased coverage. The nucleotide position with significant increases in coverage was set to zero. The black arrow indicates the cleavage position

After confirming that *mazF*_*DR0417*_ encodes an endoribonuclease, we investigated its sequence specificity using a modified RNA‐seq approach recently developed in our laboratory (Miyamoto et al., 2016). In this approach, five synthetic RNA constructs (1000‐1, 1000‐2, 1000‐3, 1000‐4, and 1000‐5) were digested with MazF_DR0417_, and the fragmented RNA products harboring the cleavage sites at their 5′‐ends were preferentially detected by identifying the nucleotide positions with increased coverage (Figure 2a), followed by extraction of the sequences located five bases upstream and five bases downstream of the identified nucleotides (Table S1).

As shown in Figure 2b, a strong consensus 4‐nt motif, TACA, was observed in the analysis of the overall top 15 sequences (Table S1), suggesting that UACA is the major target of MazF_DR0417_. However, RNA 500‐2 was degraded into many fragments (Figure 1b, lane 5), despite that it contains only two UACA sites (Table S2). Thus, we speculated that MazF_DR0417_, similar to other MazF homologues (Miyamoto et al., 2016; Park, Yamaguchi, & Inouye, 2011; Schifano et al., 2014; Verma & Bhatnagar, 2014; Zhu et al., 2009), possesses suboptimal cleavage sequences with relatively weak cleavage affinities. When we next analyzed the overall top 25 sequences, UACC, UACU, and AACA were additionally detected (Table S1 and Figure S2), indicating that MazF_DR0417_ recognizes these specific sequences with lower affinities. In all reference samples, coverage increased at the second adenine (UACA, UACC, UACU, and AACA) (Table S1). Accordingly, MazF_DR0417_ was likely to recognize these tetrads and cleave RNAs immediately upstream of the adenine (Figures 2b and S2).

### 3.3 MazF_DR0417_ cleaves specific tetrads in a strict manner

To further confirm that MazF_DR0417_ is a sequence‐specific endoribonuclease, we utilized a fluorescence quenching technique. Briefly, short oligonucleotides tagged with 6‐carboxyfluorescein and black hole quencher‐1 at the 5′‐end and 3′‐end, respectively, were treated with endoribonucleases. Because the two dyes were tethered by DNA and/or RNA nucleotides, the fluorescence of 6‐carboxyfluorescein was typically quenched. However, as oligonucleotides were cut and unbound, their fluorescence intensities increased because of the increasing amount of unquenched 6‐carboxyfluorescein (Miyamoto et al., 2016, 2016; Wang & Hergenrother, 2007). We evaluated the cleavage specificity of MazF_DR0417_ using the oligonucleotides listed in Table 1.

**Table 1:** Sequences of fluorescently labeled oligonucleotides

Name	Sequence (5′ to 3′) a
DR‐14‐UACA	AAAAAUACAAAAAA
DR‐14‐UACC	AAAAAUACCAAAAA
DR‐14‐UACU	AAAAAUACUAAAAA
DR‐14‐AACA	AAAAAAACAAAAAA
D‐13‐AAA	AAAAAAAAAAAAA
R‐13‐GUUGU	GUUGUCAUGCCGG
R‐13‐UCUCG	UCUCGGUGCGUUG
R‐13‐UGACA	UGACACGAACCGC
DR‐14‐CACA	AAAAACACAAAAAA
DR‐14‐UAUA	AAAAAUAUAAAAAA
DR‐13‐ACA	AAAAAACAAAAAA

a Underlined letters represent RNA nucleotides, whereas other letters represent DNA nucleotides.

**Figure 3:**
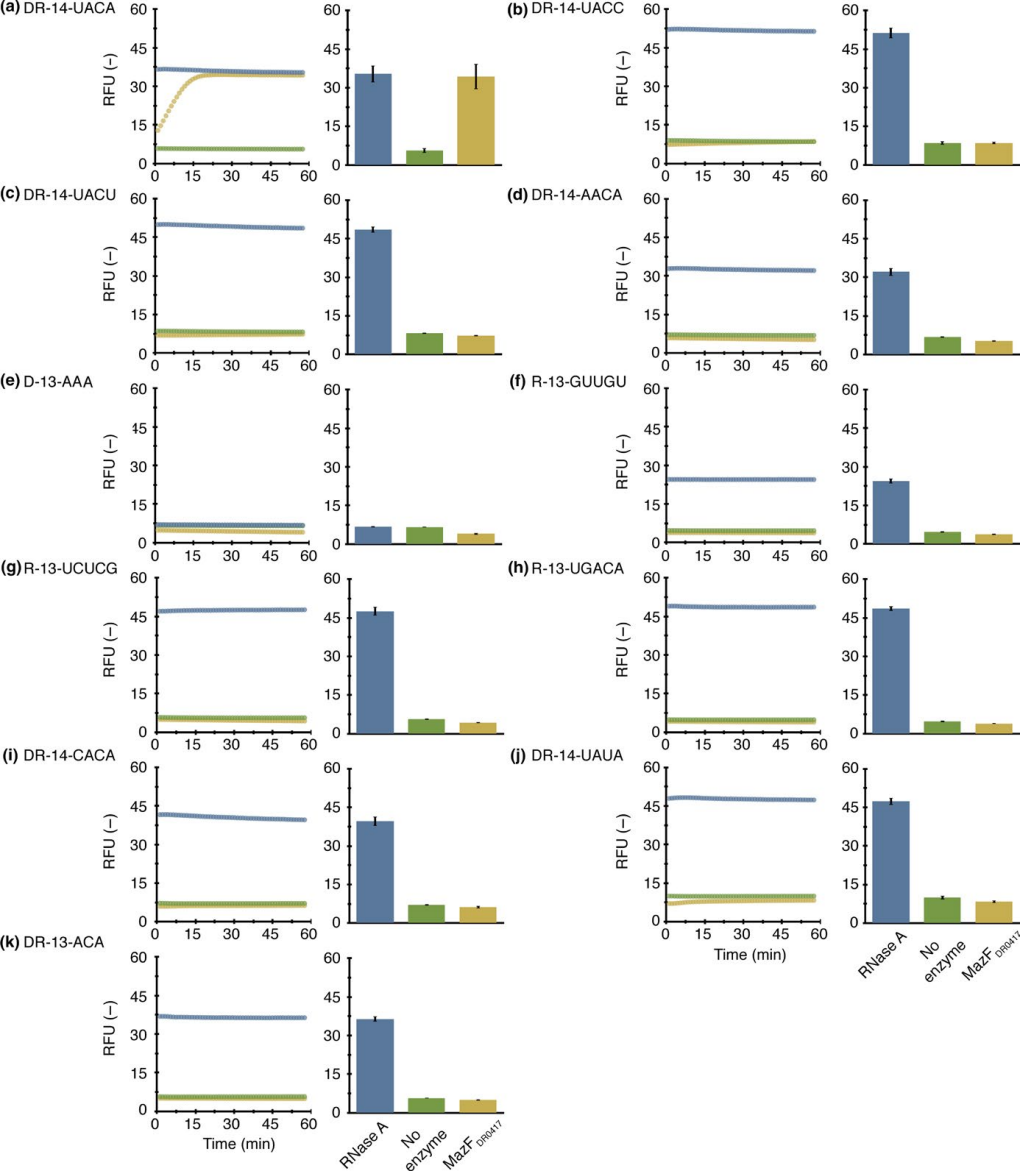
Sequence‐specific RNA cleavage with a small amount of MazF_DR__0417_. Fifteen nanograms of MazF_DR__0417_ (yellow) was incubated with 10 pmol of fluorescently modified oligonucleotides: (a) DR‐14‐UACA, (b) DR‐14‐UACC, (c) DR‐14‐UACU, (d) DR‐14‐AACA, (e) D‐13‐AAA, (f) R‐13‐GUUGU, (g) R‐13‐UCUCG, (h) R‐13‐UGACA, (i) DR‐14‐CACA, (j) DR‐14‐UAUA, and (k) DR‐13‐ACA. In the control reactions, fluorescence intensities in the presence of 100 ng of RNase A (blue) and absence of enzymes (green) at each time point (left) and the end point (right) were measured

**Figure 4:**
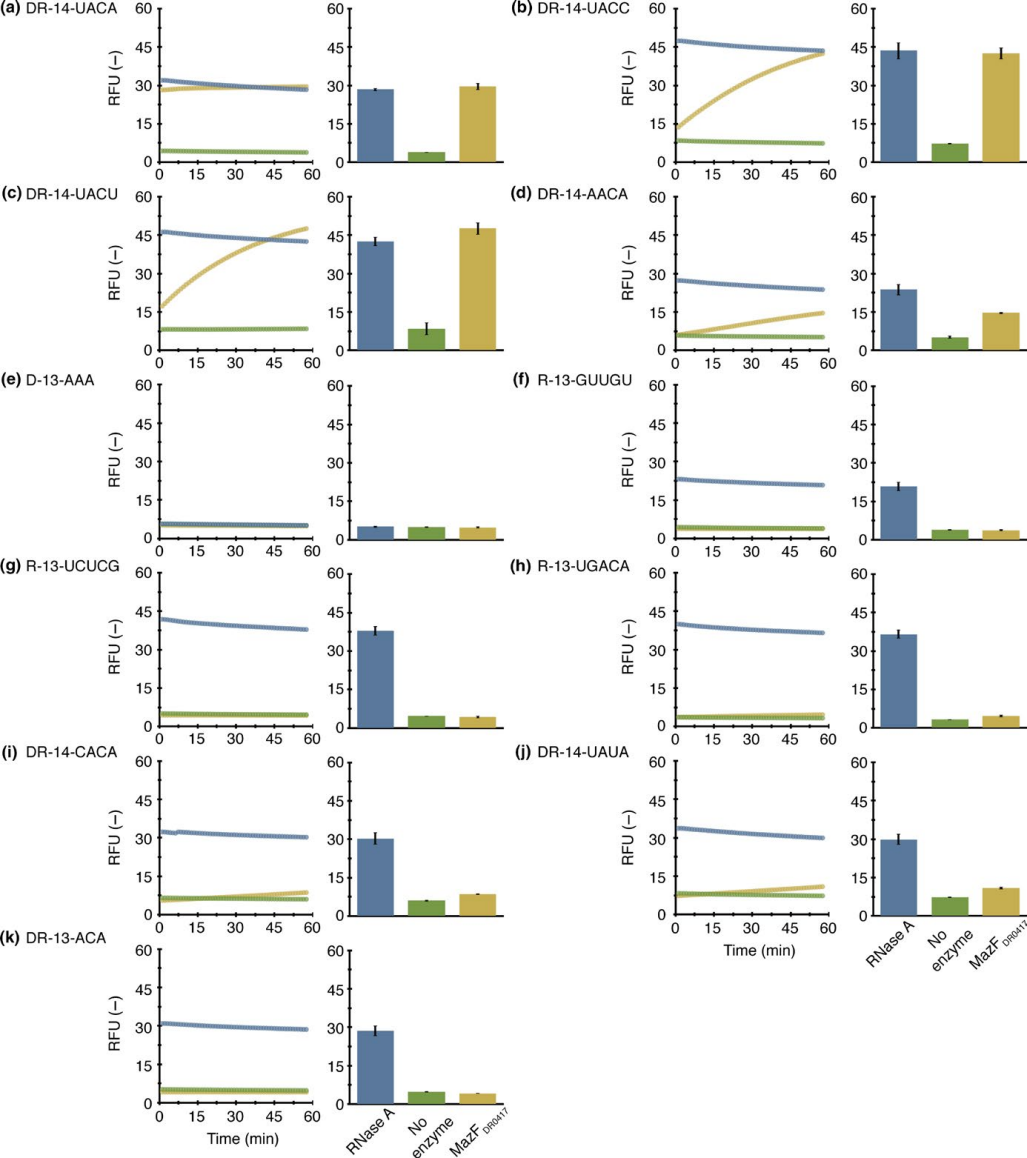
Sequence‐specific RNA cleavage with an excessive amount of MazF_DR__0417_. Six hundred nanograms of MazF_DR__0417_ (yellow) was incubated with 10 pmol of fluorescently modified oligonucleotides: (a) DR‐14‐UACA, (b) DR‐14‐UACC, (c) DR‐14‐UACU, (d) DR‐14‐AACA, (e) D‐13‐AAA, (f) R‐13‐GUUGU, (g) R‐13‐UCUCG, (h) R‐13‐UGACA, (i) DR‐14‐CACA, (j) DR‐14‐UAUA, and (k) DR‐13‐ACA. In the control reactions, fluorescence intensities in the presence of 100 ng of RNase A (blue) and absence of enzymes (green) at each time point (left) and the end point (right) were measured

To examine whether UACA and three other sequences (UACC, UACU, and AACA) are the determinants of MazF_DR0417_‐mediated RNA cleavage, we synthesized DNA‐RNA chimeric oligonucleotides including corresponding RNA tetrads (DR‐14‐UACA, DR‐14‐UACC, DR‐14‐UACU, and DR‐14‐AACA). Consistent with the RNA‐seq results, DR‐14‐UACA was cleaved (Figures 3a and 4a). Meanwhile, the three other chimeric oligonucleotides (DR‐14‐UACC, DR‐14‐UACU, and DR‐14‐AACA) were cleaved less effectively, as cleavage was not observed until an excessive amount of MazF_DR0417_ was added (Figures 3b–d and 4b–d). These results reinforce that UACA is the major cleavage site of MazF_DR0417_.

Notably, there was no increase in fluorescence intensity in a DNA oligonucleotide (D‐13‐AAA) associated with MazF_DR0417_ treatment (Figures 3e and 4e). Furthermore, none of the three RNA oligonucleotides (R‐13‐GUUGU, R‐13‐UCUCG, and R‐13‐UGACA), the internal sequences of which were identical to that of the RNA substrate (RNA 1000‐4) used in the RNA‐seq, were cleaved by MazF_DR0417_ (Figures 3f–h and 4f–h). Because these oligonucleotides are devoid of specific tetrads, these results support the sequence specificity of MazF_DR0417_.

Finally, to investigate whether MazF_DR0417_ recognition is strict, we prepared three mutated oligonucleotides (DR‐14‐CACA, DR‐14‐UAUA, and DR‐13‐ACA). Because the RNA‐seq results suggested that MazF_DR0417_ cleaves RNAs at U^ACA (where ^ denotes the position of the cleavage), we altered the bases preceding and following the second adenine to the pyrimidine bases C and U, respectively (DR‐14‐CACA and DR‐14‐UAUA). In addition, an oligonucleotide whose first uracil was removed from the tetrad was synthesized (DR‐13‐ACA). In all cases, we observed complete blockage (Figures 3i–k) or reduction in cleavage (Figures 4i–k), demonstrating that the recognition of MazF_DR0417_ is specific to some tetrads. From these data, we concluded that MazF_DR0417_ is a four‐base cutter whose prime target is UACA.

## 4 DISCUSSION

TA systems are common in prokaryotic chromosomes and plasmids and are frequently found in multiple loci in the same organism (Chopra et al., 2013; Pandey & Gerdes, 2005; Sevin & Barloy‐Hubler, 2007). They are activated under stressful conditions and they enhance bacterial stress resistance. Depending on the antitoxin nature and its manner of neutralizing toxin activity, TA systems are currently classified into six distinct classes (Page & Peti, 2016). Among these, the type II TA system, where a protein antitoxin inhibits the activity of its cognate protein toxin by forming a toxin‐antitoxin complex, is one of the most widely studied classes (Schuster & Bertram, 2013). Based on the similarities in the toxin sequences, these systems are further divided into several families (i.e., MazEF, VapBC, and HigBA) (Leplae et al., 2011).

In the current study, we demonstrated that a *D. radiodurans* chromosomal TA pair encoded by the DR0416 and DR0417 loci forms a canonical type II MazEF system (Figures 1b and S1b). Furthermore, we revealed that the MazF toxin (MazF_DR0417_) functions as a 4‐nt specific cutter and strictly recognizes the UACA tetrad (Figures 2, 3, 4). To the best of our knowledge, this is the first MazF that specifically recognizes the U^ACA sequence (Masuda & Inouye, 2017; Schifano & Woychik, 2017). Recently, Zorzini et al., (2016) determined the crystal structure of *E. coli* MazF (MazFec) in complex with the substrate analogue d(A^1^U^2^ A ^3^ C ^4^ A ^5^U^6^A^7^), and reinforced the notion that MazFec recognizes ACA triplet strictly and that MazFec cleaves the substrate at the position of ^ACA and A^CA (Miyamoto et al., 2016; Vesper et al., 2011; Zhang, Zhang, Hara, Kato, & Inouye, 2005a; Zhang et al., 2003). They mentioned that the MazFec recognition site could be divided into two different regions: first, the one where dU^2^ is located, which is called the upstream binding site; and second, the one that accommodates d(A^3^C^4^A^5^U^6^), which is called the downstream binding groove. In the upstream region, they observed a prominent cavity, and they reasoned that both purine and pyrimidine bases could be accommodated in the space; thus, any base could be located at the position of one‐base upstream of the first A (ACA). Furthermore, they mentioned that MazFec possesses two distinct positions of the cleavage sites (^ACA and A^CA), as the lack of hydrogen bonds between dU^6^ in the downstream binding groove and the MazFec recognition site reduces the specificity of the cleavage position. Taken together, MazF_DR0417_ is distinct from MazFec in the following two points: first, it recognizes the unprecedented tetrad, UACA; indeed it does not cleave ACA (Figures 3k and 4k). Second, MazF_DR0417_ cleaves U^ACA (Figure 2b).

It was predicted that the gene encoded at DR0662 also codes for a MazF toxin (MazF_DR0662_) (Pandey & Gerdes, 2005). Given that these two MazF endoribonucleases show only 22.8% similarity (Figure S3), it is not surprising that they recognize distinct sequences. In fact, although the cleavage sequence and recognition length of MazF_DR0662_ remain unknown, Shimada, Takayama, Asada, & Kato, (2011) previously suggested that purified MazF_DR0662_ cleaved the RNA oligonucleotide lacking the UACA tetrad at the UU^CCUUU site. Therefore, the difference in cleavage specificity may be beneficial for *D. radiodurans* to enrich specific transcripts to withstand certain environmental stresses.

Sequence‐specific toxin endoribonucleases have been proposed to target messenger RNAs selectively, resulting in protein‐mediated RNA interference; therefore, these endoribonucleases were also referred to as “mRNA interferases” (Christensen‐Dalsgaard & Gerdes, 2008; McKenzie et al., 2012; Zhang, Yamaguchi, & Inouye, 2009; Zhang et al., 2005b; Zhang, Zhu, Zhang, & Inouye, 2005b; Zhang et al., 2003; 2005 Zhu et al., 2006). However, recent studies documented that some of these endoribonucleases also target transfer RNA, enabling cells to alter their translation using a different mechanism (Cruz et al., 2015; Schifano et al., 2016). In these reports, single‐stranded regions of tRNAs were cleaved by endoribonucleases, thereby blocking protein synthesis. Analysis of tRNA sequences of *D. radiodurans* revealed that the UACA sequence was located within the anticodon stem‐loop of tRNA^Val^ (Figure S4a and Table S2). Thus, MazF_DR0417_ may also inhibit translation indirectly by inactivating the function of tRNA^Val^ as an adaptor molecule; indeed, MazF_DR0417_ halved the chemically synthesized tRNA^Val^ *in vitro* (Figure S4b). Future studies are necessary to validate whether native tRNA^Val^ is a genuine target of this enzyme.

In summary, our data showed that MazF_DR0417_ is a functional endoribonuclease and recognizes a unique tetrad, UACA. These data indicate that this enzyme enables *D. radiodurans* to acclimate to environmental changes through direct and/or indirect growth modulation.

## CONFLICTS OF INTEREST

The authors declare no conflict of interest.
