# Supplementary material for: Characterization of a Deinococcus radiodurans MazF: A UACA‐specific RNA endoribonuclease
Source: Microbiologyopen. 2017 Jul 3;6(5):e00501. doi: 10.1002/mbo3.501 (PMC5635168; doi:10.1002/mbo3.501)
Supplement: Supplementary file 1 [file MBO3-6-na-s001.pdf]

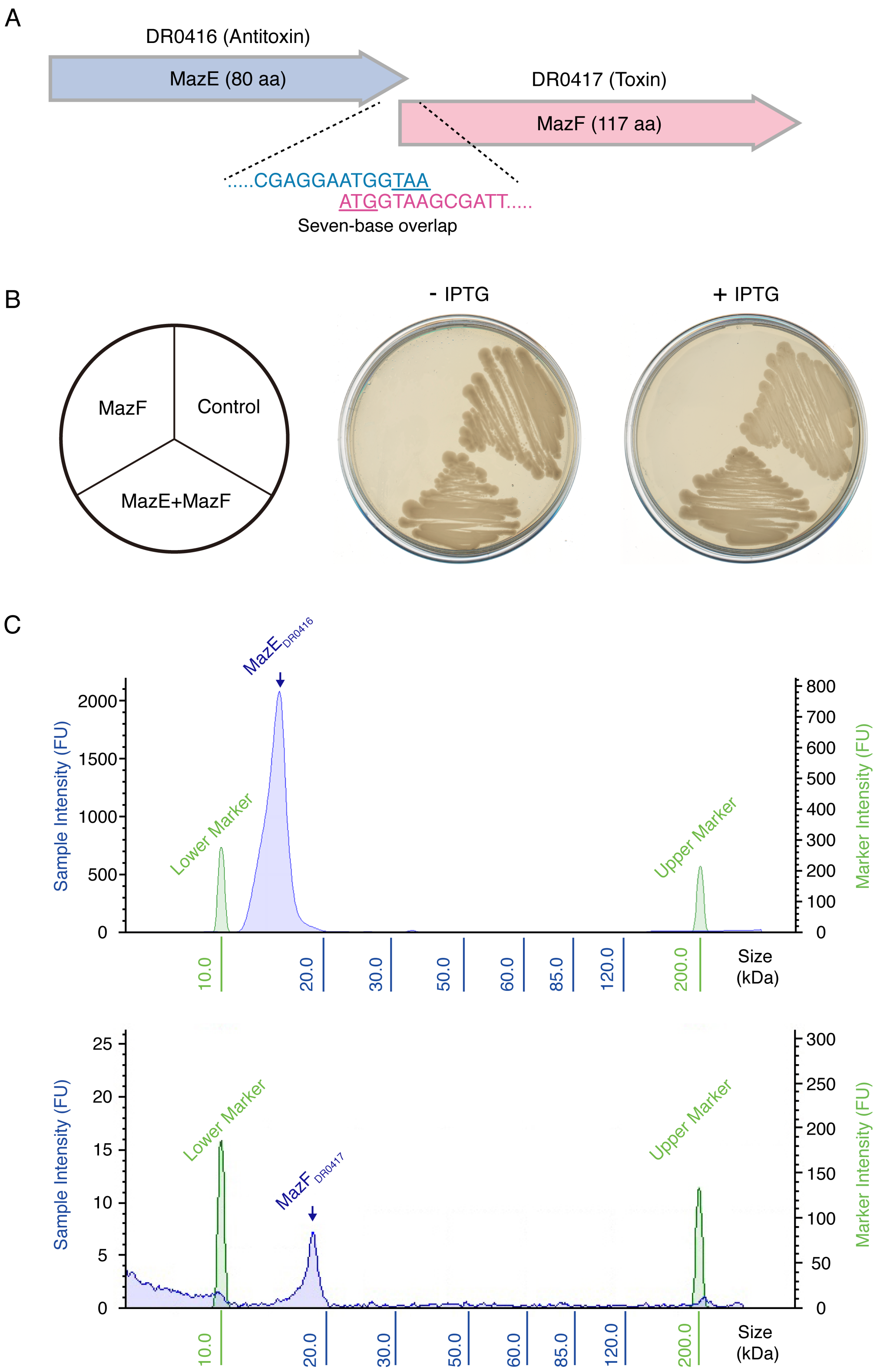

Figure S1. A MazEF homologue conserved in *D. radiodurans*. (A) Schematic representation of MazEF pair. A part of gene sequences of *mazE*$_{\mathrm{DR0416}}$ and *mazF*$_{\mathrm{DR0417}}$ were shown in blue and pink, respectively. Underlined letters indicate the stop codon of *mazE*$_{\mathrm{DR0416}}$ and start codon of *mazF*$_{\mathrm{DR0417}}$, respectively. (B) *D. radiodurans* MazEF expression in *E. coli*. BL21(DE3) cells were transformed with pET21c empty plasmid, pET21c encoding *D. radiodurans mazF* or *mazEF*. The cells were streaked onto the LB (0.2% glucose, 100 μg/mL ampicillin) plate in the presence of (right pannel) or in the absence of 25 μM IPTG (middle pannel). (C) Molecular weight and purity of isolated MazE$_{\mathrm{DR0416}}$ (upper panel) and MazF$_{\mathrm{DR0417}}$ (lower panel).

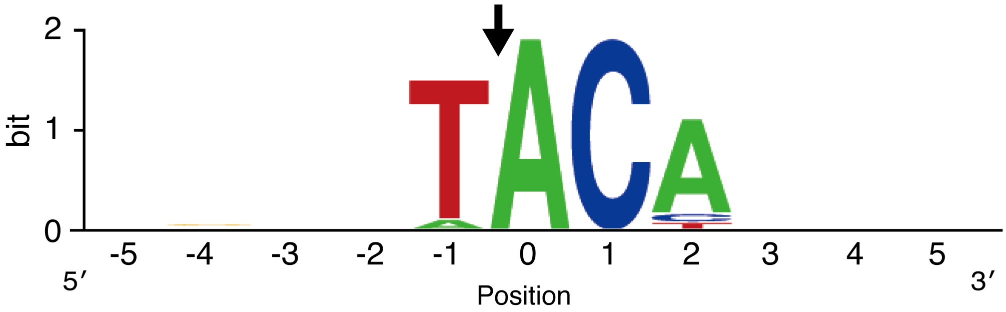

Figure S2. Potential cleavage sites of $MazF_{DR0417}$. Conserved sequences around the nucleotide positions with increased coverage. Overall top 25 sequences were analyzed using WebLogo. The nucleotide position with significant increases in coverage was set to zero. The black arrow indicates the position of the cleavage.

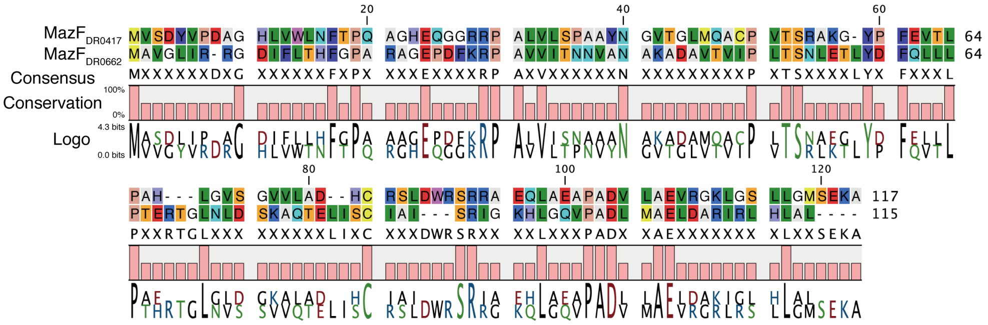

Figure S3. Pairwise alignment of two MazF sequences conserved in *D. radiodurans*.

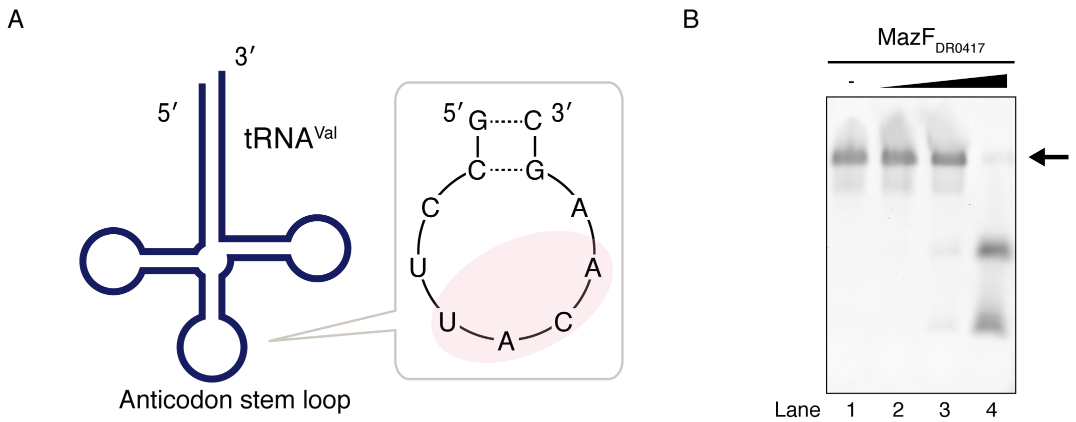

Figure S4. Cleavage of the chemically synthesized tRNA. (A) Schematic representation of $tRNA^{Val}$. (B) $MazF_{DR0417}$-mediated tRNA cleavage. Lane 1, control reaction without any enzyme; lanes 2–4, reaction with 0.1, 0.9, and 8.1 pmol of $MazF_{DR0417}$ added, respectively. The black arrow indicates the full length of $tRNA^{Val}$.

Table S1 Extracted 25 sequences with $\mathrm{MazF}_{\mathrm{DR0417}}$ cleavage

Rank	RNA type	Position	Relative coverage increase	Coverage	Sequence (5′ to 3′) [a]
1	1000-3	719	1069.22	9,623	AGCGTACATTC
2	1000-2	109	211.98	9,963	ACCATACAACC
3	1000-2	434	124.50	5,478	GGCTTACAGTG
4	1000-5	592	115.98	9,162	CCCATACAACC
5	1000-5	831	30.28	18,167	GTGGTACAAAT
6	1000-5	186	25.16	5,208	CAGGTACAATG
7	1000-5	385	21.11	8,255	TAGATACACTC
8	1000-2	901	18.27	1,279	CGTTTACACCG
9	1000-5	89	15.44	2,239	TGACTACACGG
10	1000-4	482	13.92	724	TGAATACACGT
11	1000-4	593	11.65	9,527	GCGGTACACAC
12	1000-5	792	9.04	624	CGTTTACATCA
13	1000-1	380	7.41	12,276	TAAGTACACGA
14	1000-2	541	4.14	9,614	GGTCTACAGCT
15	1000-4	220	3.47	2,862	TTACTACAGGC
16	1000-4	577	2.98	855	CGCGTACATTT
17	1000-1	200	1.86	5,642	AACGTACAGCG
18	1000-4	89	1.80	983	TGCTTACCCTC
19	1000-2	547	1.79	17,425	CAGCTACAGGG
20	1000-1	177	1.40	2,970	CGACAACACCG
21	1000-1	837	1.37	3,720	CTATTACTTCG
22	1000-1	64	1.32	1,604	GCTGTACCTAA
23	1000-5	264	1.30	6,581	CGCGTACAGAC
24	1000-3	190	1.30	918	GAGGTACTTAG
25	1000-4	883	1.28	1,085	CCATAACATAA

[a] Underlined letters represent the base with significant coverage increase

Table S2 RNA sequences used in this study

Name	Sequence (5′ to 3′) [a, b]
RNA 500-2	GGGAGACUAAAUCUCGGCGUCGGUUCAUACGCGCGAUCGUUUGCUGUCAGGGC<br>AUACUCGAAUCCGGACUCCGACAAUUAUAGGCCAUCCUGAAUAGCCGAUCAUGC<br>GAGUCACGAUAAGGCAGGCUCUGCGAUAUCCCGAUAUACUGGAGAAGCUGAAUC<br>CCACCUAGAGCGAACUGUCAGAGGAUCGACCUCAGGCUCGCUAUCAUCAUAACG<br>GCGGACGACCUGUGUCACAUUCCGAACGCUACGUGACGAUAUUAUCUGUCGAAA<br>GGCAUAGAACGCCGGUCAAUAUCCUGCGGCAUUCUCUUUAUCACCGGCUAUAAC<br>UACUAGGUUCCGCAGAUAUAGACUGCGCACGGAACAUGUAGAUAGAUCGAGUAG<br>GGUAGCGAUUUAACGACUCGACU**UACA**GACAGAGACGUAGAACGUCAGACGAGU<br>GGUAUGCCCACCAGAGGCGA**UACA**GGCUGUACCUGCGUAGCACUAGAGUCGUG<br>CGUCAUGCGGACCCUAUCUAAAAAAAAAAAAAAAAAAAAAAAAAAAAAA
$tRNA^{Val}$	GGGCGAUUAGCUCAGCGGUAGAGCGCUCGCCU**UACA**AGCGAUUGGUCGGGGGU<br>UCAAAUCCCUCAUCGCCCA

[a] The UACA sequence is shown in bold letters. [b] The other three tetrads detected with RNA-seq are underlined with a solid line
